# Nectar Characteristics and Honey Production Potential of Five Rapeseed Cultivars and Two Wildflower Species in South Korea

**DOI:** 10.3390/plants13030419

**Published:** 2024-01-31

**Authors:** Sung-Joon Na, Young-Ki Kim, Ji-Min Park

**Affiliations:** Department of Forest Bioresources, National Institute of Forest Science, Suwon 16631, Republic of Korea; treeace@korea.kr (Y.-K.K.); bright86@korea.kr (J.-M.P.)

**Keywords:** nectar, sugar content, amino acid composition, honey production

## Abstract

The growing beekeeping industry in South Korea has led to the establishment of new honey plant complexes. However, studies on honey production from each species are limited. This study aimed to assess the honey production potential of various *Brassica napus* cultivars and two wildflower species. The nectar characteristics of *B. napus* varied significantly among the cultivars. Absolute sugar concentrations differed among the cultivars, but sugar composition ratios were similar. In contrast, the amino acid content remained relatively uniform regarding percentage values, irrespective of the absolute concentrations. Estimations of honey potential production per hectare (kg/ha) resulted in the following ranking among cultivars: ‘JM7003’ (107.1) > ‘YS’ (73.0) > ‘JM7001’ (63.7) > ‘TL’ (52.7) > ‘TM’ (42.4). The nectar volume of *Pseudolysimachion rotundum* var. *subintegrum* and *Leonurus japonicus* increased during the flowering stage. *P. rotundum* var. *subintegrum* was sucrose-rich and *L. japonicus* was sucrose-dominant. Both species predominantly contained phenylalanine, *P. rotundum* var. *subintegrum* had glutamine as the second most abundant amino acid, and *L. japonicus* had tyrosine. The honey production potential was 152.4 kg/ha for *P. rotundum* var. *subintegrum* and 151.3 kg/ha for *L. japonicus*. These findings provide a basis for identifying food resources for pollinators and selecting plant species to establish honey plant complexes.

## 1. Introduction

### 1.1. Ecological Significance and Economic Implications of Honey Plants

Honey and various bee products have advantages attributed to their potential positive effects on human health. As a result, honey has been utilized for nutritional and therapeutic purposes since ancient times [1,2,3]. The viability of the apiculture industry depends on factors including the bee species, environmental conditions, and botanical origin [4]. Flowering plants provide food resources, such as nectar and pollen, to honeybees, and, reciprocally, honeybees assist in the pollination of these flowering plants, aiding their reproduction and survival [5]. This symbiotic relationship forms the basis of the apiculture industry, which includes beekeeping products and vital crop pollination services. Therefore, the maintenance and expansion of flowering plants are crucial for the sustainable development of the apiculture industry. This ensures an adequate supply of food for honeybees, allowing them to pollinate entomophilous plants and continue facilitating crop production [6]. Preserving and increasing the diversity and abundance of flowering plants is vital for maintaining ecological balance and supporting the health and reproduction of pollinators such as honeybees [7,8]. Consequently, this contributes to the long-term sustainability of the apiculture industry.

Diverse and abundant nectar plants have a positive effect on the population growth of plants and insects [9,10]. Conversely, the scarcity of nectar plant resources has led to a reduction in honeybee product yield and a decline in wild pollinators [11,12]. Recently, there have been decreases in both the diversity and abundance of pollinators worldwide [13,14,15,16]. Multiple factors, such as global climate change, altered land use, pesticides, diseases, and insufficient nutrition, contribute to the decline [17,18,19,20,21,22,23,24,25]. With the increasing recognition of the ecological contributions of floral resources, various methods have been proposed to expand floral resources and enhance service functions across agricultural ecosystems [26,27,28].

According to statistics from the Ministry of Agriculture, Food, and Rural Affairs (MAFRA), South Korea, the beekeeping industry has been consistently growing, with the number of beekeeping farms increasing from 19,000 in 2011 to 29,000 in 2020, a 152% growth over the past decade. The number of bee colonies increased by 175% during the same period. However, despite this quantitative growth, the average annual natural honey production decreased from 25,000 tons in 2007–2011 to 13,000 tons in 2016–2020 [29]. This situation is the result of a complex interplay of various factors, including the nationwide synchronized flowering plants due to climate change. However, a scarcity of floral resources has been identified as the primary cause. Currently, in South Korea, efforts are being made to promote the development of the beekeeping industry and increase the income of beekeeping farms. The “Beekeeping Industry Promotion and Support Act” was enacted in 2020 to protect and distribute honey plants. Large-scale honey plant complexes are being established in various regions with financial support from the government. Article 2 of the Enforcement Rules encourages the cultivation of honey plants by specifying a range of honey plants, including 25 woody and 15 herbaceous plant species. However, studies on the apicultural aspects of honey plant valuation, including nectar secretion, honey production potential, and sugar and amino acid contents of each honey plant, are lacking.

Not all flowering plants are equally important to the apiculture industry. Each plant differs in nectar production capacity and sugar composition, both quantitatively and qualitatively [30]. Moreover, the proportion of secreted nectar utilized by honeybees varies among these honey plants. Recently, studies have been conducted to estimate honey production per unit area by considering growth characteristics, flowering quantity, and nectar characteristics (nectar volume and sugar content) for each honey plant species [30,31,32]. These studies provide valuable information for establishing honey plant complexes based on plants with superior honey productivity at the regional and national levels [26]. Importantly, the evaluation of plants for the establishment of honey plant complexes at the regional or national levels must be conducted using consistent methods.

Sugars and amino acids in nectar are essential energy sources for honey production and bee colony development. Therefore, the sugar and amino acid profiles of plants are valuable factors that explain the relationship between pollinators and their source plants [33,34]. The composition of nectar varies depending on the plant taxa [30,32], environmental conditions in which the plant grows [35], floral sexual phases [36], and flower position within inflorescences [37]. The quality and composition ratio of sugars in nectar, as well as the absolute amount per flower, can significantly influence the appeal of plants to pollinators. The profitability of flowers is a key determining factor in the selection of plants that bees visit to obtain food [38]. However, the quantity of sugars and amino acids in the floral nectar from specific plants can contribute to ‘flower constancy’ or ‘pollinator constancy’ [39]. Amino acids are important attractors that determine plant visits by pollinators [40,41] and are key nutrients for many consumers [42,43]. Amino acids activate specific taste chemoreceptors and stimulate or inhibit sensory cell responses [44,45,46]. Thus, they attract pollinators and protect plants from herbivores [47,48]. Specific amino acid concentrations provide information to insects regarding the intensity of nectar taste [48], and pollinators prefer nectar with a specific amino acid composition [49]. Therefore, a better understanding of host plants is crucial for bee production, as well as sustainable bee management. These findings are likely to provide a basis to identify food resources for pollinators and select plant species to establish honey plant complexes.

There are approximately 625 species of honey plants in Korea, including herbaceous and woody plants [50]. The plant species tested in this study, *Brassica napus* L. (rapeseed), *Pseudolysimachion rotundum* var. *subintegrum* T. Yamaz (mountain spike speedwell), and *Leonurus japonicus* Houtt. (motherwort), are included in this list and have been used as honey plants for a long time. However, quantitative evaluations of honey plant value have not been conducted, and only honeybee foraging behavior has been observed. Therefore, this study aimed to elucidate the value of honey plants for five recommended varieties of *B. napus* in Korea, *P. rotundum* var. *subintegrum*, and *L. japonicus*, which have not yet been globally studied for their honey plant characteristics. Accordingly, we applied a consistent methodology to determine nectar secretion, the sugar content and composition, and the quantitative and qualitative compositions of amino acids. In addition, we estimated the potential honey production per plant per hectare by considering plant growth characteristics. 

### 1.2. Research Plants: Utilization and Significance

*B. napus* is a crucial crop in temperate regions and ranks as one of the four major global oil crops, along with soybeans and peanuts [51,52]. It accounts for approximately 13–16% of global vegetable oil production [53]. In addition to its use in edible oils, *B. napus* is utilized for producing high-quality animal feed and biodiesel [54]. Because of its abundant flowering in early spring, *B. napus* is a valuable food resource for various insects until other plants start blooming after winter. While approximately 70% are known to be self-fertile or autogamous [55], visits from pollinating insects still provide benefits by increasing the seed yield [56,57]. *B. napus* flowers develop two pairs of nectaries: the lateral (inner) and median (outer) pairs. It was believed that median nectaries did not produce nectar. However, subsequent studies have revealed that nectar is produced in the median nectaries and that anatomical differences are associated with differences in nectar production [55]. Moreover, Davis et al. [58] reported that nectar produced from lateral nectaries had a higher quantity and showed a higher glucose/fructose ratio (1.0–1.2) compared to those from median nectaries (0.2–0.9). The differing nectar production capabilities of nectaries led most bees to visit the lateral nectaries when foraging on *B. napus* flowers [59]. Bee activity for collecting nectar and pollen appears to be greater in the afternoon (12:00–2:00 p.m.) than in the morning [57].

*P. rotundum* var. *subintegrum* was previously categorized as belonging to the genus *Veronica*. However, based on molecular phylogenetic studies, it is now classified within the genus *Pseudolysimachion* [60,61,62]. *P. rotundum* var. *subintegrum* thrives naturally throughout Korea because of its excellent ecological adaptability [63]. Its popularity as an ornamental plant is attributed to being perennial; it has long blooming periods and is easy to maintain [64]. Currently, the detailed pharmacological effects specific to *P. rotundum* var. *subintegrum* have not been extensively documented. However, considering that extracts from many species within the Plantaginaceae family exhibit various pharmacological actions, including antioxidant, anticancer, antimicrobial, and anti-inflammatory activities, it is anticipated that *P. rotundum* var. *subintegrum* could become a valuable crop in the future [65,66,67,68].

*L. japonicus* is a perennial herb belonging to the Lamiaceae family and is widely distributed in East Asia, including Korea, China, and Thailand. It typically blooms from July to August. It has traditionally used to treat gynecological and obstetrical diseases, as indicated by its name, and is beneficial to women [69]. More than 280 secondary metabolites have been isolated from this plant, showing various activities, such as anticoagulant [70], antibacterial [71,72], antiplatelet aggregation [73,74,75], vasodilation [76], angiogenesis [77], and effects on uterine smooth muscle [78].

## 2. Results

### 2.1. Comparison among B. napus Cultivars 

#### 2.1.1. Flowering and Growth Characteristics

The flowering periods of the five cultivars of *B. napus* sown on the same date varied slightly (Table 1). ‘JM7003’ bloomed the earliest, starting on 25 May and flowering for a total of 20 days until June 13. ‘YS’ flowered from 27 May to 13 June, whereas ‘TM’, ‘YL’, and ‘JM7001’ all began flowering on May 30 and concluded flowering by June 16. ‘JM7001’ finished flowering on June 20. Plant height was tallest in ‘JM7003’ at 73.1 cm, followed by ‘JM7001’ (65.3 cm), ‘TL’ (60.7 cm), ‘TM’ (54.7 cm), and ‘YS’ (53.4 cm) in descending order. The number of flowers per plant was the highest in ‘JM7003’ at 65.3, followed by ‘JM7001’ with 43.7 flowers per plant. ‘TL’, ‘TM’, and ‘YS’ had 27.2 to 33.2 flowers per main stem, with the maximum and minimum numbers differing approximately twofold. Plant density per unit area (m^2^/plants) was relatively high for ‘YS’ and ‘JM7001’ and relatively low for ‘TM’ and ‘TL’, and no statistically significant difference was observed between the cultivars. These data were used to quantify potential honey production per unit area.

#### 2.1.2. Nectar Secretion and Sugar Composition

The secreted nectar volume per flower over the lifespan (1 day) was the highest in ‘JM7003’ (1.54 μL/flower), followed by ‘YS’ (1.08 μL/flower), ‘JM7001’ (1.04 μL/flower), ‘TL’ (0.81 μL/flower), and ‘TM’ (0.73 μL/flower) in descending order (*p* = 0.005).

The free sugar content differed significantly among the cultivars for all constituents (sucrose, glucose, and fructose). The total sugar content was highest in ‘TM’ (1349.4 μg/μL) and lowest in the ‘JM7003’ (641.0 μg/μL). Overall, ‘TM’, ‘TL’, and ‘YS’ exhibited higher sugar contents per unit volume, whereas ‘JM7001’ and ‘JM7003’ showed slightly lower sugar contents per unit volume. These results were consistent in the sugar components as well, with the ‘JM7003’ and ‘JM7001’ having lower quantitative amounts of sucrose, glucose, and fructose compared to the other cultivars (Table 2). 

The floral nectar of *B. napus* consists primarily of glucose and fructose, with sucrose constituting a very low proportion, ranging from 1.2% to 1.9% of the total sugar content. Glucose accounted for 47.6–49.5% of the total sugar content, while fructose constituted 48.9–50.7% of the sugar composition. Notably, the sugar composition was more uniform than the absolute quantities. Sucrose and glucose compositions showed no differences among the cultivars. Regarding fructose, the ‘JM7003’ had the highest content at 50.7%, whereas the ‘JM7001’ had the lowest content at 48.9%, but the difference was marginal. 

**Figure 1:**
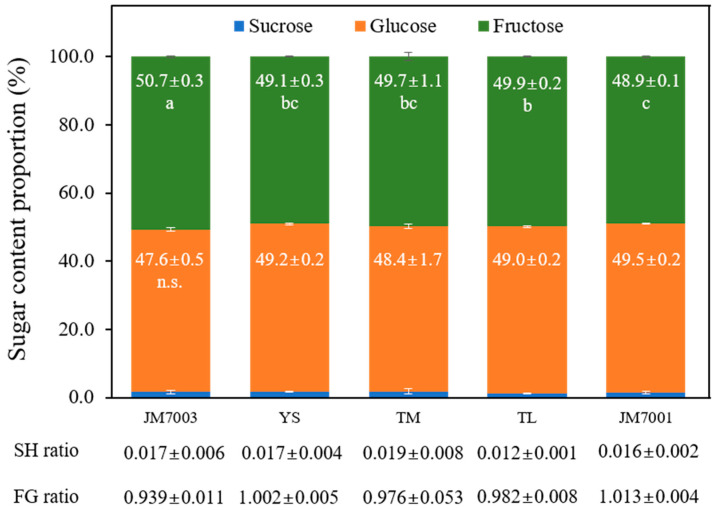
Sugar composition in the floral nectar of five *B. napus* cultivars. Data were subjected to one-way ANOVA with post hoc comparisons using Duncan’s multiple range test at the 5% level. Different letters in each column indicate statistical differences, and n.s. means non-significance.

The sucrose to hexose ratio (SH ratio) showed a slight difference, with ‘TM’ at 0.019 and ‘TL’ at 0.012. The fructose to glucose ratio (FG ratio) varied among cultivars, with ‘JM7001’ at 1.013 and ‘JM7003’ at 0.939, but the differences were not statistically significant (Figure 1).

#### 2.1.3. Amino Acid Content

**Figure 2:**
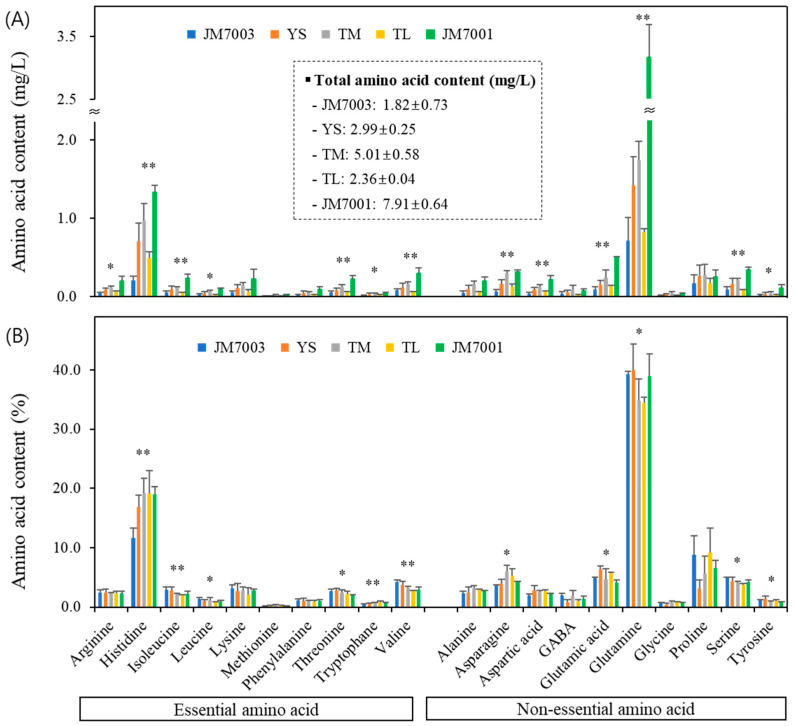
Amino acid contents of five *B. napus* cultivars. Results are expressed as either absolute concentrations (**A**) or the composition of the total amino acid content (**B**). Data were subjected to one-way ANOVA with post hoc comparisons using Duncan’s multiple range test at the 5% level. * and ** indicate significant differences between cultivars at *p* < 0.05 and <0.01, respectively.

In *B. napus* nectar, 20 different amino acids were identified, and significant variability was observed in their absolute concentrations. In quantitative terms, ‘YS’ had the highest content at 7.91 mg/L, followed by ‘TM’ (5.01 mg/L), ‘JM7001’ (2.99 mg/L), ‘TL’ (2.36 mg/L), ‘JM7003’ (1.82 mg/L), showing significant differences among the cultivars (Figure 2A). Among all cultivars, glutamine had the highest absolute concentration, and considerable variability was observed for both glutamine and histidine. The glutamine absolute value for ‘YS’ was 3.2 mg/L, which was over four times higher than that of ‘JM7003’ (0.7 mg/L) and ‘TL’ (0.8 mg/L). The second most abundant amino acid in all cultivars was histidine, with a difference of approximately sevenfold between ‘YS’ and ‘JM7003’.

The amino acid composition ratio showed that glutamine (34.4–40.0%) and histidine (11.6–19.2%) were the most abundant in all cultivars (Figure 2B). On average, proline (5.6–9.2%), glutamic acid (4.1–6.6%), and asparagine (3.6–6.0%) had relatively high composition ratios, with variations in rank among the cultivars. For example, proline was the third most abundant in ‘JM7003’, ‘JM7001’, and ‘TL’, but in ‘TM’, asparagine had a higher proportion, and in ‘YS’, glutamic acid was more abundant than proline. The amino acids constituting more than 5% of the nectar composition were proline in both ‘JM7003’ (8.8%) and ‘JM7001’ (6.6%) and glutamic acid (6.3%) in ‘YS’. Additionally, in ‘TM’, asparagine (6.0%) and proline (5.6%) were present in concentrations exceeding 5%. For ‘TL’, proline (9.2%), glutamic acid (5.8%), and asparagine (5.2%) were present in concentrations exceeding 5%. Serine was present in all cultivars at a concentration of over 3% (3.7–4.9%), whereas the amino acids with a less than 1% composition were glycine (0.5–0.7%), tryptophan (0.5–0.8%), and methionine (0.1–0.3%). Histidine, isoleucine, leucine, threonine, tryptophan, valine, asparagine, glutamic acid, glutamine, serine, and tyrosine exhibited statistically significant differences in composition ratios among the cultivars (*p* < 0.05).

The average essential amino acid content in the floral nectar of the five *B. napus* cultivars was 33.5%. ‘JM7003’ had the lowest content at 30.3%, whereas the other four cultivars showed similar contents ranging from 33.3% to 35.6%.

#### 2.1.4. Estimated Honey Production

The nectar sugar content per flower, calculated as the product of nectar volume and free sugar content, was highest in ‘YS’ at 1.24 mg/flower, followed by ‘TM’ and ‘JM7003’ at 0.96 mg/flower, ‘TL’ at 0.95 mg/flower, and ‘JM7001’ with the lowest content at 0.80 mg/flower. Despite noticeable differences, no statistically significant differences were observed among the cultivars (Table 3).

However, by estimating the potential honey production per plant by multiplying the nectar sugar content per flower and the number of flowers per plant, the honey production was highest in ‘JM7003’ at 72.0 mg per plant among the five cultivars. ‘YS’ and ‘JM7001’ followed at 45.3 and 40.2 mg, respectively. ‘TL’ and ‘TM’ had honey potentials of 36.3 and 29.9 mg, respectively. Furthermore, by calculating the potential honey production per hectare using the number of plants per hectare, ‘JM7003’ exhibited excellent honey productivity at 107.1 kg/ha, followed by ‘YS’ at 73.0 kg. The estimated honey production per hectare for ‘JM7001’ and ‘TL’ were 63.7 and 52.7 kg, respectively. ‘TM’ showed the lowest honey production at 42.4 kg.

### 2.2. Two Wildflowers

#### 2.2.1. Flowering and Growth Characteristics

The flowering and growth characteristics of *P. rotundum* var. *subintegrum* and *L. japonicus* are presented in Table 4. The flowering period of *P. rotundum* var. *subintegrum* lasted for 41 days, from July 19 to August 30. The average plant height was 83 cm, and the average number of flowers per plant was 3422. Motherwort flowered for 32 days from August 2 to September 2. The average plant height was 124.8 cm, with an average of 2894 flowers per plant. The planting spacing was 30 × 25 cm, resulting in a plant density of 17.5 plants/m^2^ for both species per unit area.

#### 2.2.2. Nectar Secretion Characteristics

Nectar volume was measured on each day of flowering because the two species of wildflowers flowered for two days (Table 5). The nectar secretion per flower of *P. rotundum* var. *subintegrum* was 0.07 μL on the first day of flowering and increased to 0.30 μL on the second day of flowering, showing an accumulation of nectar as flowering progressed (*p* = 0.0099). The free sugar content per unit volume of nectar slightly decreased as flowering progressed, with 828.7 μg/μL on the first day and 767.9 μg/μL on the second day. On the first day of flowering, the absolute quantities of sucrose were 407.9 μg/μL and glucose and fructose were 197.7 and 232.2 μg/μL, respectively. On the second day of flowering, the composition of nectar sugars was as follows: sucrose was 310.2 μg/μL, glucose was 211.2 μg/μL, and fructose was 246.5 μg/μL. The free sugar content per flower, calculated using nectar secretion and the free sugar content per unit volume, was 0.06 mg on the first day of flowering and significantly increased to 0.22 mg on the second day of flowering (*p* = 0.0015). 

The nectar volume per flower of *L. japonicus* was 0.16 μL on the first day of flowering and increased to 0.33 μL on the second day of flowering, showing a significant difference between the flowering days (*p* = 0.0057). The free sugar content per unit volume was 775.4 μg/μL on the first day and 816.3 μg/μL on the second day, with no significant difference. On the first day of flowering, sucrose was 624.7 μg/μL, glucose was 71.8 μg/μL, and fructose was 78.9 μg/μL. On the second day of flowering, sucrose was 631.1 μg/μL, whereas glucose and fructose were 88.9 and 96.3 μg/μL, respectively. The free sugar content per flower was 0.12 and 0.26 mg on the first and second days of flowering, respectively, indicating a higher value on the second day (*p* = 0.0106).

#### 2.2.3. Sugar Content and Composition

**Figure 3:**
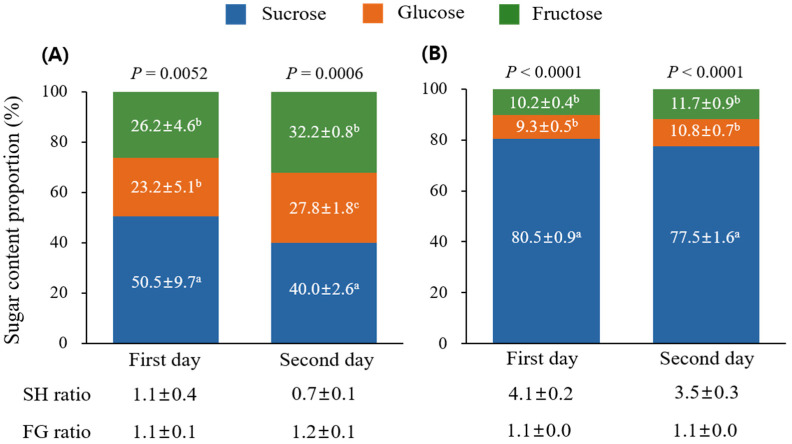
Sugar composition in collected floral nectar from *P. rotundum* var. *subintegrum* (**A**) and *L. japonicus* (**B**). Data were subjected to one-way ANOVA with post hoc comparisons using Duncan’s multiple range test at the 5% level. Different letters in each column indicate statistical differences.

The sugar content in *P. rotundum* var. *subintegrum* showed that sucrose was the most abundant sugar, followed by fructose and glucose, on both the first day of flowering (*p* = 0.0052) and the second day of flowering (*p* = 0.0006). As flowering progressed, the proportion of sucrose decreased (first day of flowering, 50.5%; second day of flowering, 40.0%), whereas the amounts of glucose and fructose slightly increased. The glucose content increased from 23.2% on the first day of flowering to 27.8% on the second day, whereas the fructose content increased from 26.2% on the first day to 32.2% on the second day. Therefore, the sucrose-to-hexose (glucose + fructose) ratio decreased from 1.1 on the first day of flowering to 0.7 on the second day. The fructose-to-glucose ratio increased from 1.1 on the first day of flowering to 1.2 on the second day, indicating a higher proportion of glucose (Figure 3).

Similarly, the nectar sugar composition in *L. japonicus* showed that sucrose was the predominant sugar on all flowering days (*p* < 0.0001), with glucose and fructose constituting approximately 10% of the sugars. Changes in sugar content per flowering day indicated a reduction in sucrose from 80.5% on the first day to 77.5% on the second day, whereas glucose and fructose contents increased by approximately 1.5%. Consequently, the sucrose-to-hexose ratio decreased from 4.1 on the first day to 3.5 on the second day, while the fructose-to-glucose ratio remained constant at 1.1, reflecting a proportional increase in glucose and fructose. 

#### 2.2.4. Amino Acid Content

**Figure 4:**
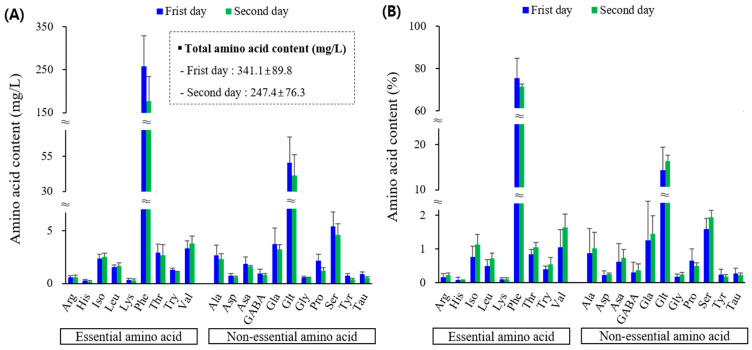
Amino acid contents as absolute concentrations (**A**) and composition as percent values of the total amino acid content (**B**) of *P. rotundum* var. *subintegrum*. Explanations: Arg—arginine, His—histidine, Iso—isoleucine, Leu—leucine, Lys—lysine, Phe—phenylalanine, Thr—threonine, Try—tryptophan, Val—valine, Ala—alanine, Asp—asparagine, Asa—aspartic acid, Gla—glutamic acid, Glt—glutamine, Gly—glycine, Pro—proline, Ser—serine, Tyr—tyrosine, Tau—taurine.

The floral nectar of *P. rotundum* var. *subintegrum* contained twenty amino acids (Figure 4). The absolute quantity of total amino acids decreased by 93.8 mg/L from 341.1 mg/L on the first day of flowering to 247.4 mg/L on the second day as flowering progressed. The content of the nine essential amino acids was an average of 230.5 mg/L, with 270.9 mg/L on the first day of flowering and 190.2 mg/L on the second day of flowering. Notably, there were increases in valine (+0.45 mg/L), isoleucine (+0.15 mg/L), leucine (+0.11 mg/L), and arginine (+0.02 mg/L), despite this overall decrease. Phenylalanine was overwhelmingly abundant among all amino acids (73.4%), and this decrease was pronounced. Specifically, it decreased significantly from 186.1 mg/L on the first day of flowering to 75.0 mg/L on the second day, indicating a reduction of 80.9 mg/L. However, when expressed as a percentage, the rate of decrease was minimal at −1.16%. The total content of 11 non-essential amino acids in quantity decreased by 13.1 mg/L from 70.2 mg/L on the first day of flowering to 57.1 mg/L on the second day as flowering progressed. However, the proportion of the overall amino acids increased from 20.6% on the first day of flowering to 23.2% on the second day of flowering. Although the proportions were small, proline (−0.16%), tyrosine (−0.07%), and taurine (−0.05%) decreased in their respective proportions. The remaining 19 amino acids accounted for 0.02–1.59% of the total content, which was very low.

**Figure 5:**
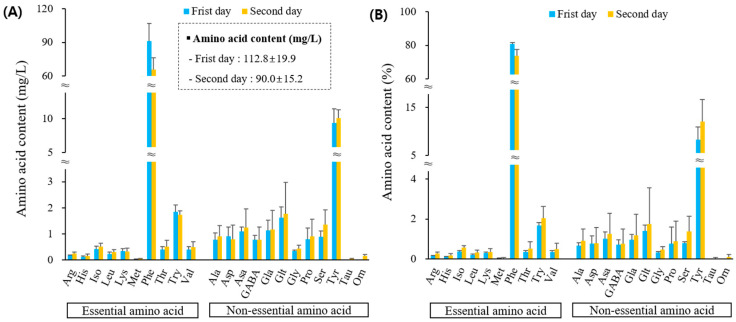
Amino acid contents as absolute concentrations (**A**) and composition as percent values of the total amino acid content (**B**) of *L. japonicus*. Explanations: Arg—arginine, His—histidine, Iso—isoleucine, Leu—leucine, Lys—lysine, Met—methionine, Phe—phenylalanine, Thr—threonine, Try—tryptophan, Val—valine, Ala—alanine, Asp—asparagine, Asa—aspartic acid, Gla—glutamic acid, Glt—glutamine, Gly—glycine, Pro—proline, Ser—serine, Tyr—tyrosine, Tau—taurine, Orn—ornithine.

The floral nectar of *L. japonicus* contained twenty-two amino acids (Figure 5). The total absolute quantity decreased from 112.8 mg/L on the first day of flowering to 90.0 mg/L on the second day, a reduction of 22.8 mg/L (−20.2%). The decrease in phenylalanine levels was particularly notable as flowering progressed. Additionally, apart from lysine, asparagine, tryptophan, GABA, and other amino acids increased on the second day of flowering compared with the first day. The proportion of the 10 essential amino acids was 81.4% (day 1: 84.3%; day 2: 78.5%), with phenylalanine being the most abundant (first day: 80.8%; second day: 73.7%). Among the 13 non-essential amino acids, tyrosine was the most abundant, slightly increasing in composition from 8.3% on the first day to 12.0% on the second day of flowering. The remaining 20 amino acids were present in very small amounts, ranging from 0.01–0.59%. Ornithine and taurine were not detected on the first day of flowering but were found in trace amounts on the second day of flowering. 

#### 2.2.5. Estimated Honey Production

Based on the sugar content per flower and number of flowers per plant in *P. rotundum* var. *subintegrum*, it is estimated that approximately 870.6 mg of honey can be produced per plant. In addition, assuming that 175,000 plants are cultivated per hectare, it is estimated that approximately 152.4 kg of honey can be produced (Table 6). 

Using the sugar content of 0.22 mg per flower and the total number of flowers per plant (2894 flowers) for *L. japonicus*, the potential honey production per plant was estimated to be 846.6 g. Extrapolating this to a planting density of 175,000 plants per hectare, the estimated honey production per hectare is 151.3 kg.

## 3. Discussion

### 3.1. Estimation of Honey Production

Significant variability was observed in the nectar volumes and absolute sugar concentrations among the different *B. napus* cultivars. Among the five *B. napus* cultivars, ‘JM7003’ recorded the highest nectar volume (1.54 μL), which was more than twice that of ‘TM’ (0.73 μL). Such significant variability has been reported in other studies. Bertazzini and Forlani [79] reported nectar volumes ranging from 0.2 μL to 0.75 μL among 44 cultivars, whereas Pierre et al. [80] documented volumes ranging from 0.7 μL to 5.9 μL among 71 genotypes. An important consideration is that the volume and concentration of nectar can vary depending on the temperature and relative humidity at the time of nectar collection [81,82]. For example, under high-temperature and low-relative humidity conditions, the moisture content of nectar decreases, reducing the absolute quantity and increasing the concentration. Conversely, under low-temperature and high-relative humidity conditions, the absolute quantity of nectar increases, lowering the nectar concentration. Therefore, when assessing honey production among species or cultivars, it is essential to consider the quantity of nectar per flower as well as various factors such as the nectar content per unit volume and the number of flowers per plant [30,32]. In this study, ‘JM7003’ had the highest nectar volume, but it had a lower free sugar content per unit volume compared to ‘YS’, which had the highest sugar content per flower (Table 3). In contrast, ‘TM’ had the lowest nectar volume, but it had the highest sugar content per unit volume, resulting in ‘TM’ having the same sugar content per flower as ‘JM7003’ (Table 2 and Table 3). However, ‘YS’, with the highest sugar content per flower, exhibited a lower number of flowers per plant, lowering overall honey production per plant compared to ‘JM7003’. Conversely, ‘JM7001’, despite having the lowest sugar content per flower, demonstrated a higher honey production per plant compared to ‘TM’ and ‘TL’ due to its higher flower count. Furthermore, it is important to consider the per hectare plant count because the currently measured flower number per plant reflects the planting density. In conclusion, among the *B. napus* cultivars developed in Korea, ‘JM7003’ exhibited the highest honey potential production (107.1 kg), followed by ‘YS’ (73.0 kg), ‘JM7001’ (63.7 kg), ‘TL’ (52.7 kg), and ‘TM’ (42.4 kg) in descending order.

Utilizing indigenous plants with high regional adaptability is advantageous for establishing and maintaining honey plant complexes in various regions and countries. *P. rotundum* var. *subintegrum* and *L. japonicus* demonstrate superior honey production potential compared to *B. napus*, which is widely used in many countries (Table 5). Additionally, these plants offer promising valuable medicinal resources (as mentioned in the Introduction). This study is the only investigation of nectar characteristics and honey production in *P. rotundum* var. *subintegrum* and *L. japonicus*. Nectar secretion in *P. rotundum* var. *subintegrum* varied significantly between the first and second days of flowering, with 0.07 μL on the first day and 0.30 μL on the second day. A similar trend was observed in *L. japonicus*. Considering that our nectar collection time was 4:00 p.m., it is evident that more nectar was secreted on the second day of flowering, irrespective of nectar reabsorption [83]. Therefore, to accurately measure nectar volume using centrifugation, collecting the cumulative nectar over the entire flower lifespan is necessary. However, the estimated range of honey production per hectare, based on the minimum and maximum native plant counts, was broad for *P. rotundum* var. *subintegrum*, ranging from 92 to 244 kg, and for *L. japonicus*, ranging from 69 to 235 kg. The existence of variability implies the possibility of breeding to increase honey production by selecting individuals with abundant flowering numbers. The medicinal use of *L. japonicus* can be categorized into two methods: harvesting before flowering during early summer and utilizing the fruits in autumn [84]. If a management approach is adopted in which honey is harvested in summer (July) and fruits are collected in autumn (October), the economic value (land productivity) per unit area may increase.

The results of this study are expected to provide valuable information in terms of offering more food resources to pollinators, as well as selecting plant species to establish honey plant complexes aimed at increasing honey production. However, it is expected that actual honey production will be lower than the estimated honey production potential in this study, as a significant portion of nectar is typically used for brood rearing and sustaining bee colonies [30].

### 3.2. Sugar Composition

The sugars in the floral nectar mainly comprise sucrose, glucose, and fructose. In some plants, small amounts of mannose, arabinose, xylose, galactose, sorbitol, maltose, and melibiose are occasionally included [79,85,86]. The sugar concentration and quality of the floral nectar are closely related to the type and frequency of pollinators [85,87]. The preference of various pollinators for these specific plants affects seed production and crop yield of the plant [88].

The sugar composition of honey is categorized as sucrose-dominant (over 1.0; sucrose 51–100%), sucrose-rich (0.5–1.0; 34–50%), hexose-rich (0.1–0.5; 10–33%), and hexose-dominant (below 1.0; 0–9%) based on the sucrose-to-hexose ratio [86,89]. In this study, all *B. napus* cultivars belonged to the hexose-dominant category (0.012–0.019) (Figure 1), which was consistent with previous studies [55,58,79,80,90,91].

The SH ratio of the floral nectar changed daily during blooming (Figure 3). On the first day of flowering in *P. rotundum* var. *subintegrum*, floral nectar was categorized as sucrose-dominant (1.1), whereas on the second day of flowering, it became sucrose-rich (0.7). In the case of *L. japonicus*, the nectar on all flowering days was sucrose-dominant, but the value decreased from 4.1 to 3.5. The increase in hexoses in the secreted nectar over time can be explained by the hydrolysis of sucrose by invertase [92], where glucose and fructose are typically present in a 1:1 ratio [93]. However, a difference existed in the sugar conversion rates between the two plants. On the first day of blooming, the sucrose content in *P. rotundum* var. *subintegrum* rapidly decreased from 50% to 40%, whereas in *L. japonicus*, it decreased by only 3%, from 80.5% to 77.5%. This indicates that the rate of sugar conversion is not the same for all plants. It can vary depending on sugar metabolism pathways and secretion processes occurring in the nectaries. This can also be a result of the presence or speed of catalytic reactions by transglucosidases and transfructosidases located in nectaries [79,94]. However, further research on this topic is required.

Honeybees show a preference for floral nectar, a blend of various sugars (sucrose, glucose, and fructose), to nectar composed only of sucrose [95,96]. However, in terms of a single sugar content, there was a tendency of sucrose > glucose > maltose > fructose [97]. Furthermore, the preference of long- or short-tongued bees and other pollinators may vary based on the sugar content [98,99,100,101,102,103,104]. However, the types of pollinators visiting plants that secrete nectar are not solely based on the sugar composition. Different types of pollinators visit plants with similar sugar compositions; however, different types of pollinators are observed [105,106,107,108].

In this study, although there were no collected data, observations were made that *Apis mellifera* visits were mostly observed in *B. napus*, where hexoses were overwhelmingly abundant in the nectar. Although sucrose was dominant, both glucose and fructose were present in high proportions in *P. rotundum* var. *subintegrum* and *L. japonicus*, resulting in diverse insect visits and making it difficult to pinpoint specific insects that lead to pollination.

Generally, floral nectar contains sugars that are essential for the survival of pollinators, along with small amounts of amino acids and secondary metabolites. The quantity and sugar composition of secreted nectar vary across species [109,110,111]. In addition, nectar contains secondary compounds such as alkaloids, phenolic substances, and iridoid glycosides, which can either increase or decrease the frequency and duration of pollinator visits [112,113]. Therefore, the type and frequency of pollinators visiting flowers can vary depending on the qualitative and quantitative composition of floral nectar, the quality and content of pollen [114], the presence and function of toxins [115,116], and the food resource characteristics provided to insects. Furthermore, the type and frequency of pollinators visiting flowers can vary based on the distance between plants and beehives, competition with other insects, and the influence of the bee colony’s intrinsic needs. Therefore, comprehensive research is required to thoroughly investigate these aspects.

### 3.3. Amino Acid Content

Amino acids play a crucial role in determining the taste of nectar and influencing pollinator visits [117]. The composition of amino acids, both in type and quantity, varies among different plant species and even within the same species [118,119]. The uptake of variety amino acids has been noted to positively influence the lifespan and fecundity of bees [120], with positive effects on memory and learning abilities [121]. In the present study, both rapeseed and mountain spike speedwell contained 20 amino acids each, whereas motherwort contained 22 amino acids, suggesting a diverse supply of amino acids to pollinators.

The amino acid contents among the different *B. napus* genotypes showed significant differences. However, when expressed as relative percentages, they still exhibited statistically distinct yet more consistent relative values (Figure 2). A similar trend was observed for sugar contents among the different genotypes (Table 2 and Figure 1). Regarding these findings, Bertazzini and Forlani [79] reported that all genotypes shared a mechanism that controls the reciprocal ratios of sugar and amino acid compositions. All rapeseed cultivars exhibited high proportions of glutamine (34.8–40.0%; avg. 37.5%) and histidine (11.6–19.2%; avg. 17.2%). Additionally, proline (3.2–8.8%; avg. 6.7%), glutamic acid (3.9–5.8%; avg. 5.2%), asparagine (3.6–6.0%; avg. 4.6%), and serine (3.7–4.9%; avg. 4.2%) exhibited relatively high compositions (Figure 2). This is consistent with the results of a previous study that investigated the amino acid compositions of 44 different genotypes of *B. napus* var. *oleifera* [79].

de Groot [122] reported 10 essential amino acids for bees that cannot be synthesized within their bodies and must necessarily be obtained from food sources. In this study, the essential amino acid contents in five *B. napus* cultivars ranged from 30.3% to 35.6%, with non-essential amino acids (64.4–69.7%) being the most abundant. However, *B. napus* is recognized as an attractive plant for bees because it can obtain additional necessary amino acids and proteins from the nectar of rapeseed flowers. Bees obtain a nutritionally balanced amino acid profile by foraging for nectar and pollen from rapeseed flowers [79,123]. However, other studies have suggested a more complex relationship between the amino acid content of nectar and bee preferences. The nutritional balance of essential amino acids and carbohydrates preferred by adult worker honey bees varies with age. Forager bees tend to prefer nectar with a higher carbohydrate content than essential amino acid content [34].

Proline, the third most abundant amino acid in rapeseed nectar, is one of the most preferred amino acids by honeybees [124,125,126,127]. This amino acid is believed to stimulate labellar salt receptor cells, allowing bees to perceive taste [48,128,129]. Proline is a crucial energy source for honeybee flight [125,130,131], which is rapidly metabolized during the oxidation process and releases significant amounts of ATP [132,133,134,135,136]. These characteristics of proline confer significant advantages to honeybees that travel long distances to forage [79]. This can be considered a co-evolutionary strategy by which plants produce proline-rich nectar to increase their foraging success rates [127]. Additionally, proline is a component of antimicrobial peptides present in *Apis mellifera* hemolymph, aiding in the removal of bacteria without damaging cell membranes [137]. Therefore, proline is a crucial amino acid providing innate immunity for honey bees [138,139,140].

Serine is also part of a serine protease enzyme involved in the immune processes of *A. mellifera* and plays a role in protecting insects from harmful microorganisms and xenobiotics [141,142]. *B. napus* nectar contains a significant amount of serine, approximately 4% (ranking sixth in overall abundance), and it is relatively abundant in *P. rotundum* var. *subintegrum* and *L. japonicus* at approximately 2%. Typically, honeybees tend to avoid nectar with a high serine content [125,126,127]. Honeybee respond negatively to glycine, leucine, valine, threonine, alanine, aspartic acid, and methionine [41]. Glutamic acid, which constitutes approximately 5% of *B. napus* nectar, is an essential donor of amide groups (glutamine and asparagine), which are crucial for the production of purine nucleotides and serotonin [143,144]. Glutamic acid and hydroxyproline are the primary determinants of the qualitative variation in amino acid composition within *B. napus* nectar and serve as major sources of nitrogen, carbon, and energy for insects [133,145,146]. Furthermore, this amino acid, along with aspartic acid (2.0–2.8%; avg. 2.4%) and lysine (2.1–3.2%; avg. 2.7%), reduces oxidative stress in *A. mellifera* larvae and adults [147,148].

This study is the first to describe the amino acid profiles of the nectar of *P. rotundum* var. *subintegrum* and *L. japonicus*. Phenylalanine, constituting over 70% of the nectar of *P. rotundum* var. *subintegrum* and *L. japonicus* (present in *B. napus* nectar from the five cultivars at around 1%), along with proline, stimulates chemosensors in forager bees, directing their focus towards collecting nectar. Hendriksma et al. [126] reported that phenylalanine showed a stronger preference than other amino acids in their analysis of amino acid preference in bees. Bees were observed to find phenylalanine highly attractive to such an extent that they would forgo 84 units of sucrose to collect 1 unit of phenylalanine. In addition, Petanidou et al. [41] found that phenylalanine was the most abundant amino acid and preferred by flower visitors. In various studies, phenylalanine has been identified as a phagostimulant that attracts honeybees to flowers, and its presence, along with tryptophan and alanine, enhances the foraging preference of honeybees [126,149]. These amino acids are crucial for pollination ecology [150]. In our study, the tryptophan content in *B. napus* nectar was approximately 0.5–0.8% (average 0.7%), while it was approximately 0.5% in *P. rotundum* var. *subintegrum* and 1.9% in *L. japonicus*. Alanine was found to be present in canola at a level of 2.4–3.1%, whereas in *P. rotundum* var. *subintegrum* and *L. japonicus*, it was approximately 0.9% and 0.8%, respectively (Figure 2, Figure 4 and Figure 5). 

The floral nectar of *P. rotundum* var. *subintegrum* and *L. japonicus* contained taurine and ornithine (Figure 4 and Figure 5). These amino acids, classified as non-protein amino acids (NPAAs), are relatively uncommon in floral nectars, but have been found in other plants as well [151,152,153,154]. Taurine is estimated to be involved in the development of flight muscles in winged insects. GABA works synergistically with taurine to suppress excessive and potentially disruptive states of excitation under stressful conditions [43].

## 4. Materials and Methods

### 4.1. Plant Material 

**Figure 6:**
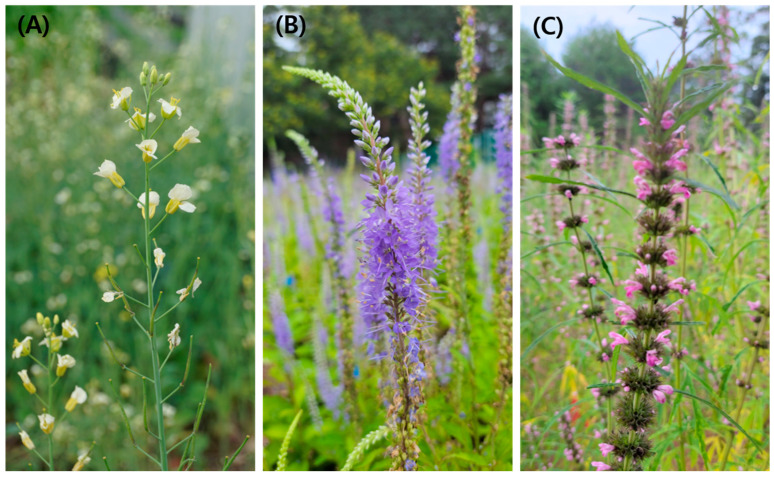
Flower and inflorescence shapes of *B. napus* ((**A**), ‘Jungmo 7003’), *P. rotundum* var. *subintegrum* (**B**), and *L. japonicus* (**C**).

This study utilized five cultivars of *B. napus*, which were developed by the National Institute of Crop Science, Rural Development Administration in South Korea. The seeds used in the research were provided by the aforementioned institute. Seeds of five *B. napus* cultivars were sown on 14 April 2022, in an experimental field located in Suwon, South Korea. *B. napus* used in this study is recommended for cultivation in South Korea. ‘Youngsan’ (‘YS’), ‘Tammi’ (‘TM’), and ‘Tamla’ (‘TL’) are developed for increased seed yield. ‘Jungmo 7001’ (‘JM 7001’) and ‘Jungmo 7003’ (‘JM 7003’) are ornamental cultivars, with ‘Jungmo 7001’ known for its large flowers and ‘Jungmo 7003’ having distinctive white flowers (Figure 6A) [155,156]. Sowing was carried out at a rate of 2 kg per 100 m^2^ according to the recommended cultivation practices in Korea.

*P. rotundum* var. *subintegrum* and *L. japonicus* were sown on 1 April 2022, after which they were transplanted at intervals of 25 × 30 cm to approximately 10 cm tall plants (Figure 6B,C). Flower counts were obtained by conducting a comprehensive survey of 50 randomly selected plants of each plant species. The number of plants per square meter (m^2^) was counted and converted to plants per hectare (ha), resulting in an estimate of potential honey production per hectare.

### 4.2. Measure of Nectar Volumes

To measure the secreted nectar volume, we selected ten healthy plant individuals with robust growth. We removed all flowers that had already bloomed and covered the entire plant with pollination bags to prevent any loss of nectar due to visits from honeybees and other insect pollinators. For plants with a 2-day flower lifespan, such as *P. rotundum* var. *subintegrum* and *L. japonicus*, we collected nectar by distinguishing the flowering time using a method involving marking flowers that had bloomed one day after the installation of pollination bags (at 2:00 p.m.) [111]. For *B. napus* plants with a one-day flowering period, we collected nectar on the same day as the installation of pollination bags at 5:00 p.m. Nectar collection from *B. napus* plants was conducted on June 3 for the ‘JM7003’ variety, which had the earliest flowering, and on June 10 for the remaining four varieties. *P. rotundum* var. *subintegrum* and *L. japonicus* were examined on August 18, respectively.

More than 50 flowers were harvested from each species and variety and placed into sterilized 50 mL centrifugal tubes equipped with a flower mesh (pore size 0.3 mm). The tubes were centrifuged at 4000 rpm for 4 min [157,158]. The collected nectar was quantified using a 50 μL micro-syringe (Hamilton Co., Reno, NV, USA). Subsequently, it was purified by adding an 80% ethanol solution at a 10-fold dilution, followed by filtration through a 0.45 μM pore centrifuge filter (Millipore, Billerica, MA, USA) to remove pollen and other impurities. Finally, the collected nectar was stored at −20 °C until further analysis of the sugar and amino acid contents.

### 4.3. Analysis of Sugar Contents

The free sugar content was analyzed using HPLC (Dionex Ultimate 3000; Dionex, Sunnyvale, CA, USA). Deionized water served as the mobile phase, flowing at a rate of 0.5 mL/min, and the oven temperature was set at 80 °C. Detection was carried out using a Shodex Ri-101 (Showa Denko, New York, NY, USA) in combination with an Aminex 87P column (300 × 7.8 mm, Bio-Rad, Hercules, CA, USA). The free sugar content was determined by the standard external method utilizing integral meter measurements, and high purity (99.5%) sucrose, glucose, and fructose (Sigma Aldrich, St. Louis, MO, USA) were employed as standards.

### 4.4. Analysis of Amino Acid Contents

The amino acids in the collected nectar samples were analyzed using OPA (O-phthalaldehyde)-FMOC (fluorenylmethyl chloroformate) derivatization. The samples were prepared by sequential mixing with borate buffer, OPA/mercaptopropionic acid (MPA), and FMOC reagent. Subsequently, analysis was conducted using HPLC 1200 series (Agilent Technologies, Santa Clara, CA, USA). The mobile phase consisted of two solutions: A solution containing 10 mM Na_2_HPO_4_ and 10 mM Na_2_B_4_O_7_·10H_2_O with a pH of 8.2, and B solution, which was a mixture of water/acetonitrile/methanol in a 10:45:45 ratio. The gradient conditions were set to change from an initial 100:0 (*v*/*v*, %) of solution A to solution B at 26–28 min, followed by 0:100 at 28–30.5 min, and maintained at 100:0 after 30.5 min. The flow rate was set to 1.5 mL/min, and an injection volume of 1 mL was used. The INNO C-18 column (150 mm × 4.6 mm, 5 μm; Youngjin Biochrom Co., Ltd., Seongnam, Republic of Korea) was maintained at a temperature of 40 °C. The UV detector was set at 338 nm, and the OPA derivative was analyzed at an emission wavelength of 450 nm and an excitation wavelength of 340 nm using a fluorescence detector. For the FMOC derivative, the emission and excitation wavelengths were set to 305 and 266 nm, respectively.

### 4.5. Honey Production Potential

The estimation of honey production and yield was calculated using the following formula, considering the surveyed nectar secretion (μL/flower), free sugar content per unit (μg/μL), number of flowers (ea/plant), and the honey potential [159]. In the final step, to enable standardization between species, we considered plant density based on crop density and calculated the population density per hectare, ultimately estimating the potential honey production per hectare (kg/ha).

Honey production (mg/plant) = nectar sugar content (mg/flower) ^1^ × number of flowers (ea/plant) × honey potential (1.15) ^2^

^1^ Nectar sugar content (mg/flower) = nectar volume (μL/flower) × free sugar content (μg/μL) × 0.001 (for unit conversion: μg to mg)
^2^ Honey potential = sugar content: honey = 85:100.

Honey yield (kg/ha) = honey production (mg/plant) × number of plants (ea/ha) × 0.000001 (for unit conversion: mg to kg).

### 4.6. Statistics

Statistical analyses were performed, including one-way analysis of variance (ANOVA) and Duncan’s multiple range test, using the integrated statistical software package SAS 8.2 (SAS Institute, Cary, NC, USA) with a significance level of 0.05. These analyses covered various aspects, including growth, nectar volume, sugar concentration (sucrose, glucose, and fructose contents), and the concentrations of individual amino acids. Additionally, a T-test was conducted to compare the nectar volume and free sugar content between different flowering dates.

## 5. Conclusions

This study investigated the honey production potential of various *B. napus* cultivars and two wildflower species. Significant variability was observed in the quantity and sugar concentration of nectar produced per flower among *B. napus* cultivars. In the comparison of *B. napus* cultivars, ‘JM7003’ exhibited the highest honey production potential, followed by ‘YS’, ‘JM7001’, ‘TL’, and ‘TM’. Utilizing cultivars with high regional adaptability could be advantageous for establishing honey plant complexes. *P. rotundum* var. *subintegrum* and *L. japonicus* showed superior honey production potential compared to *B. napus*, suggesting their potential use in future honey plant complexes. The study results, examining the composition and changes in nectar components, sugar content, and amino acid content, are expected to provide valuable information for selecting honey plant species to increase food resources for pollinators and boost honey production. However, it is anticipated that actual honey production may be lower than the estimated potential due to bees using a significant portion of honey for brood rearing and hive maintenance. Finally, the diversity in amino acid composition across different honey plant species implies the potential to attract various types of pollinators and enhance honey production.

## Figures and Tables

**Table 1:** Results of the survey on flower characteristics and plant density.

Cultivars	Flowering Period	Height (cm)	No. of Flowers (ea/plant)		Plant Density (m^2^/plant)
			Mean	Range	
‘JM7003’	5/25–6/13	73.1 ± 13.7 ^a^	65.3 ± 8.7 ^a^	49–77	148.8 ± 39.9 ^n.s.^
‘YS’	5/27–6/13	53.4 ± 7.9 ^d^	31.8 ± 7.2 ^c^	21–42	161.0 ± 31.2
‘TM’	5/30–6/16	54.7 ± 10.3 ^cd^	27.2 ± 5.3 ^c^	16–34	142.0 ± 27.4
‘TL’	5/30–6/16	60.7 ± 9.4 ^bc^	33.2 ± 10.2 ^c^	23–49	145.3 ± 9.2
‘JM7001’	5/30–6/20	65.3 ± 11.0 ^b^	43.7 ± 5.9 ^b^	36–56	158.7 ± 20.1
*p*-value	-	<0.0001	<0.0001	-	0.8829

Data represent the means ± SD. Data were subjected to one-way ANOVA with post hoc comparisons using Duncan’s multiple range test at the 5% significance level. Different letters in each column indicate statistical differences, and n.s. indicates non-significance.

**Table 2:** Nectar secretion and sugar characteristics of five *B. napus* cultivars.

Cultivars	Nectar Volume (μL/flower)	Free Sugar Content (μg/μL)			
		Sucrose	Glucose	Fructose	Total
‘JM7003’	1.54 ± 0.33 ^a^	10.1 ± 2.3 ^c^	307.5 ± 58.9 ^c^	325.3 ± 60.7 ^c^	641.0 ± 118.7 ^c^
‘YS’	1.08 ± 0.21 ^b^	19.3 ± 6.6 ^ab^	564.3 ± 122.0 ^ab^	563.2 ± 122.0 ^ab^	1146.8 ± 248.6 ^ab^
‘TM’	0.73 ± 0.18 ^b^	25.0 ± 5.0 ^a^	656.8 ± 200.6 ^a^	667.7 ± 181.0 ^a^	1349.4 ± 381.3 ^a^
‘TL’	0.81 ± 0.23 ^b^	13.5 ± 2.3 ^bc^	575.2 ± 132.2 ^ab^	585.7 ± 135.5 ^a^	1174.4 ± 269.8 ^ab^
‘JM7001’	1.04 ± 0.18 ^b^	12.3 ± 4.2 ^c^	388.8 ± 133.1 ^bc^	384.1 ± 132.2 ^bc^	785.25 ± 269.4 ^bc^
*p*-value	0.0005	0.0004	0.0062	0.0046	0.0048

Data represent the means ± SD. Data were subjected to one-way ANOVA with post hoc comparisons using Duncan’s multiple range test at the 5% significance level. Different letters in each column indicate significant differences between the groups.

**Table 3:** Estimation of honey production considering the nectar and flowering characteristics of five *B. napus* cultivars.

Honey Potential	‘JM7003’	‘YS’	‘TM’	‘TL’	‘JM7001’
Nectar sugar content ^1^	0.96 ± 0.02	1.24 ± 0.32	0.96 ± 0.24	0.95 ± 0.31	0.80 ± 0.20
Honey production per plant ^2^	72.0 (54–85)	45.3 (30–60)	29.9 (18–37)	36.3 (25–54)	40.2 (33–52)
Honey yield per hectare ^3^	107.1 (81–141)	73.0 (54–89)	42.4 (36–54)	52.7 (51–57)	63.7 (56–72)

Data represent the means ± SD, and range data are given in parentheses. ^1^ Nectar sugar content (mg/flower) = nectar volume (μL/flower) × free sugar content (μg/μL) × 0.001 (for unit conversion: μg to mg). ^2^ Honey production (mg/plant) = nectar sugar content (mg/flower) × number of flowers per plant (ea/plant) × honey potential (1.15). ^3^ Honey yield (kg/ha) = honey production (mg/plant) × number of plants per hectare (ea/ha) × 0.000001 (for unit conversion: mg to kg). Nectar volume and the free sugar content can be found in Table 2. The number of flowers and the number of plants per hectare can be found in Table 1.

**Table 4:** Results of the survey on the growth and flower characteristics of *P. rotundum* var. *subintegrum* and *L. japonicus*.

Characteristic	*P. rotundum* var. *subintegrum*	*L. japonicus*
Flowering period	19 July–30 August	2 August–2 September
Plant height (cm)	83.0 ± 13.6	124.8 ± 27.8
Number of flowers (ea/plant)	3422 ± 370 (2064–5490)	2894 ± 318 (1320–4490)
Planting density (m^2^/plant)	17.5; 30 × 25 cm	17.5; 30 × 25 cm

Data represent the means ± SD, and range data are given in parentheses.

**Table 5:** Nectar volume, free sugar content and nectar sugar content of *P. rotundum* var. *subintegrum* and *L. japonicus*.

Nectar Characteristics	Flowering Time		*t*-Test
	First Day	Second Day	
*P. rotundum* var. *subintegrum*			
Nectar volume (μL/flower)	0.07 ± 0.01	0.30 ± 0.09	*p* = 0.0114
Free sugar content (μg/μL)	828.7 ± 266.2	767.9 ± 206.8	n.s.
- Sucrose	407.9 ± 116.5	310.2 ± 103.0	n.s.
- Glucose	197.7 ± 86.0	211.2 ± 45.8	n.s.
- Fructose	232.2 ± 93.8	246.5 ± 59.9	n.s.
Nectar sugar content (mg/flower) *	0.06 ± 0.03	0.22 ± 0.02	*p* = 0.0015
*L. japonicus*			
Nectar volume (μL/flower)	0.16 ± 0.05	0.33 ± 0.01	*p* = 0.0057
Free sugar content (μg/μL)	775.4 ± 88.6	816.3 ± 139.4	n.s.
- Sucrose	624.7 ± 73.8	631.1 ± 95.1	n.s.
- Glucose	71.8 ± 8.5	88.9 ± 21.3	n.s.
- Fructose	78.9 ± 7.8	96.3 ± 23.5	n.s.
Nectar sugar content (mg/flower) *	0.12 ± 0.04	0.26 ± 0.03	*p* = 0.0106

Data represent the means ± SD. T-test between flowering times, significant at *p* = 0.05. n.s. indicates non-significance. * Nectar sugar content (mg/flower) = nectar volume (μL/flower) × free sugar content (μg/μL) × 0.001 (for unit conversion: μg to mg).

**Table 6:** Estimation of honey production considering the nectar and flowering characteristics of *P. rotundum* var. *subintegrum* and *L. japonicus*.

Honey Potential	*P. rotundum* var. *subintegrum*	*L. japonicus*
Honey production per plant ^1^	870.6 (525–1397)	864.6 (394–1341)
Honey yield per hectare ^2^	152.4 (92–244)	151.3 (69–235)

Range data are given in parentheses. ^1^ Honey production (mg/plant) = nectar sugar content (mg/flower) × flower number per plant (ea/plant) × honey potential (1.15). ^2^ Honey yield (kg/ha) = honey production (mg/plant) × number of plants per hectare (ea/ha) × 0.000001 (for unit conversion: mg to kg). Nectar sugar content per flower can be found in Table 5. The number of flowers (ea/plant) and the number of plants per hectare (m^2^/plant) can be found in Table 4.

## Data Availability

Data are contained within the article.
